# Evolutionarily new genes in humans with disease phenotypes reveal functional enrichment patterns shaped by adaptive innovation and sexual selection

**DOI:** 10.21203/rs.3.rs-3632644/v1

**Published:** 2023-11-21

**Authors:** jianhai chen

**Affiliations:** university of chicago

**Keywords:** New genes, Pleiotropy, Young genes, Phenotypic innovation, Sexual selection, Natural selection

## Abstract

New genes (or young genes) are structural novelties pivotal in mammalian evolution. Their phenotypic impact on humans, however, remains elusive due to the technical and ethical complexities in functional studies. Through combining gene age dating with Mendelian disease phenotyping, our research reveals that new genes associated with disease phenotypes steadily integrate into the human genome at a rate of ~ 0.07% every million years over macroevolutionary timescales. Despite this stable pace, we observe distinct patterns in phenotypic enrichment, pleiotropy, and selective pressures between young and old genes. Notably, young genes show significant enrichment in the male reproductive system, indicating strong sexual selection. Young genes also exhibit functions in tissues and systems potentially linked to human phenotypic innovations, such as increased brain size, bipedal locomotion, and color vision. Our findings further reveal increasing levels of pleiotropy over evolutionary time, which accompanies stronger selective constraints. We propose a “pleiotropy-barrier” model that delineates different potentials for phenotypic innovation between young and older genes subject to natural selection. Our study demonstrates that evolutionary new genes are critical in influencing human reproductive evolution and adaptive phenotypic innovations driven by sexual and natural selection, with low pleiotropy as a selective advantage.

## Introduction

The imperfection of DNA replication serves as a rich source of variation for evolution and biodiversity [1–3]. Such genetic variations underpin the ongoing evolution of human phenotypes, with beneficial mutations being conserved under positive selection, and detrimental ones being eliminated through purifying selection. In medical terminology, this spectrum is categorized as “case and control” or “health and disease,” representing two ends of the phenotypic continuum [4]. Approximately 8,000 clinical types of rare Mendelian disorders, affecting millions worldwide, are attributed to deleterious DNA mutations in single genes (monogenic) or a small number of genes (oligogenic) with significant effects [5, 6]. To date, over 4,000 Mendelian disease genes have been identified, each contributing to a diverse array of human phenotypes (https:/mirror.omim.org/statistics/geneMap) [7]. These identified genes and associated phenotypes could provide critical insights into the evolutionary trajectory of human traits [8].

Evolutionarily new genes – such as *de novo* genes and gene duplicates – have been continually emerging and integrating into the human genome throughout the macroevolutionary process of human lineage [9–15]. Previous reports revealed that human disease genes tend to be primarily ancient, with many tracing back to the last common ancestor of eukaryotes [16]. This conclusion aligns with the deep conservation of many critical biological processes shared among cells, such as DNA replication, RNA transcription, and protein translation, which emerged early in the tree of life. Consequently, it may be inferred that new genes play less or no important role in biomedical processes. However, decades of genetic studies in non-human systems have provided extensive evidence contradicting this intuitive argument. New genes can integrate into biologically critical processes, such as transcription regulation, RNA synthesis, and DNA repair [17, 18]. For instance, in yeast, some *de novo* genes (*BSC4* and *MDF1*) play roles in DNA repair process [19–21]. In *Drosophila* species, lineage-specific genes can control the key cytological process of mitosis [22]. New genes (*Nicknack* and *Oddjob*) have also been found with roles in early larval development of *Drosophila* [23]. In Pristionchus Nematodes, some lineage-specific genes could serve as the developmental switch determining mouth morphology [24]. Moreover, in multiple insect lineages, embryonic development of body plans, which was long believed to be governed by deeply conserved genetic mechanisms, was found to be driven by newly arising genes [25]. These studies from model species reveal various important biological functions of new genes.

Compared to non-human model organisms, where gene functions can be characterized through genetic knockdowns and knockouts, interrogating functions of human genes in their native context is unfeasible. Despite this limitation, numerous omics data and in vitro studies in human genes have suggested the potential roles of evolutionary young genes in basic cellular processes and complex phenotypic innovations [26–28]. Brain transcriptomic analysis has revealed that primate-specific genes are enriched among up-regulated genes early in human development, particularly within the human-specific prefrontal cortex [29]. The recruitment of new genes into the transcriptome suggests that human anatomical novelties may evolve through the contribution of new gene evolution. Recent studies based on organoid modeling also support the importance of de novo genes on human brain size enlargement [30, 31]. These lines of evidence in recent decades about the functions of new genes contradict the conventional conservation-dominant understanding of human genetics and phenotypes.

In this study, we tackled the complexities of human phenotypic evolution and the underlying genetic basis by integrating gene age dating with analyses of Mendelian disease phenotypes. As a direct indicator of functional effects, the anatomical organ/tissue/system phenotypes (OP) affected by causal genic defects can allow us to understand the influence of gene ages on phenotypic enrichment, pleiotropy, and selective constraints along evolutionary journey. We aimed to understand include whether, what, and why human anatomical/physiological/cellular phenotypes could be affected by human evolutionary new genes. Notably, disease gene emergence rates per million years were found to be similar among different macroevolutionary stages, suggesting the continuous integration of young genes into biomedically important phenotypes. Despite the consistent pace of gene integration per million years, younger disease genes, with lower pleiotropy score, display accelerated sexual selection and human-specific adaptive innovations. By contrast, older genes are higher in pleiotropic burden that impacts more anatomical systems and are thus under stronger selective constraints. These patterns suggest that new genes can rapidly become the genetic bases of human critical phenotypes, especially the reproductive and innovative traits, a process likely facilitated by their low pleiotropy.

## Results

### Ages and organ/tissue phenotypes of human genetic disease genes

**Figure 1:**
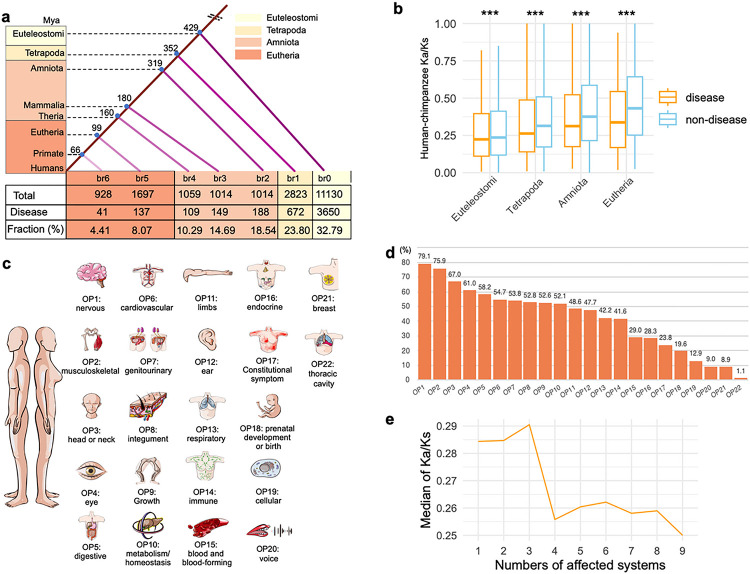
Number distribution and Ka/Ks ratios of genes categorized by ages and disease phenotypes (also organ phenotype genes). (**a**) The phylogenetic framework illustrating gene ages and disease genes associated with organ phenotypes. The phylogenetic branches represent age assignment for all genes and disease genes. The “br” values from br0 to br7 signify ancestral age groups (or branches). These are further categorized into four evolutionary age stages. The vertical axis depicts the divergence time sourced from the Timetree database (July 2023). The numbers of total genes and disease genes and their ratios are shown for each evolutionary age stage. (**b**) The pairwise Ka/Ks ratios from Ensembl database based on Maximum Likelihood estimation for “one to one” orthologs between human and chimpanzee. Only genes under purifying selection are shown (Ka/Ks < 1). The significance levels are determined using the Wilcoxon rank sum test, comparing disease genes to non-disease genes. The symbol “***” indicates significance level of *p*< 0.001. (**c**) The 22 HPO-defined organ/tissue systems, which are ordered based on the proportion of genes among all disease genes. (**d**) Percentages representing disease genes affecting various organs/tissues/systems in relation to the total number of disease genes.

We determined the ages of 19,665 non-redundant genes, following the phylogenetic framework of the GenTree database [32] and gene model annotations from Ensembl v110 (Supplementary table 1). To ensure comparable gene numbers across different age groups, we merged evolutionary age groups with a small number of genes (< 100) into their adjacent older group. As a result, we classified these genes into seven ancestral age groups, ranging from Euteleostomi (or more ancient) nodes to modern humans (br0-br6, Fig. 1a). These evolutionary groups have been further categorized into four evolutionary age epochs, starting from the oldest, Euteleostomi, to progressively younger stages of Tetrapoda, Amniota, and Eutheria, each containing over 2000 genes. Disease gene data were sourced from Human Phenotype Ontology database (HPO, Sep 2023), which is the *de facto* standard for phenotyping of rare Mendelian diseases [33]. This repository synthesizes information from diverse databases, including Orphanet [34, 35], DECIPHER [36], and OMIM [37]. An intersection of these data sets yielded 4,946 genes annotated with both evolutionary age and organ/tissue/system-specific phenotypic abnormalities (Fig. 1a and Supplementary Table 2). Contrasting earlier estimates which suggest that only 0.6% of young genes arising in Eutherian lineage could contribute to human disease genes, we observed nearly 10 times higher percentage of disease genes in this age group (6.67%, Fig. 1a and Supplementary Table 2). This indicates that the role of younger genes as disease genes might have been significantly underestimated.

To better ascertain if disease genes evolve under different evolutionary pressures compared to non-disease genes, we compared the metric of Ka/Ks ratio, which is the ratio of the number of nonsynonymous substitutions per nonsynonymous site (Ka) to the number of synonymous substitutions per synonymous site (Ks). We retrieved the “one to one” human-chimpanzee orthologous genes and the corresponding pairwise Ka/Ks ratios (12830 genes) from Ensembl database. We also evaluated whether the pattern is consistent with Ka/Ks ratios of human-bonobo and human-macaque orthologs. To include more orthologous genes, we did not use Ka/Ks ratios based on more distant species (such as the test of branch-model). Interestingly, Ka/Ks ratios were consistently lower in disease genes than in non-disease genes for human-chimpanzee orthologs (0.250 vs. 0.321), human-bonobo orthologs (0.273 vs. 0.340), and human-macaque orthologs (0.161 vs. 0.213) (Wilcoxon rank sum test, *p* < 2.2e-16 for all three datasets). These results revealed that disease genes are under significantly stronger purifying selection than non-disease genes, suggesting the important component of selective pressure in constraining the sequence evolution of disease genes. In addition, we observed an increase in Ka/Ks ratios (< 1) for genes from older to younger stages, suggesting a trend of relaxed purifying selection in young genes (Fig. 1b and Supplementary Fig. 1). Notably, despite the relaxation of purifying selection for younger genes, disease genes still tend to show lower Ka/Ks ratio than non-disease genes, suggesting a general pattern of stronger purifying selection in disease genes during evolutionary process.

We observed a heterogeneous distribution of disease genes underlying 22 HPO-defined anatomical systems, suggesting varied genetic complexity for diseases of different systems (Fig. 1c–1d). None of disease genes was found to impact all 22 systems. In contrast, 6.96% of disease genes (344/4946) were specific to a single system’s abnormality. Notably, four systems – the genitourinary system (with 81 genes), the eyes (68 genes), the ears (63 genes), and the nervous system (55 genes) – collectively represented 77.62% of these system-specific genes (267/344, Supplementary table 2). The nervous system displayed the highest fraction of diseases genes (79%, Fig. 1d). A significant 93.04% of genes were linked to the abnormalities of at least two systems (4602/4946), indicating broad disease impacts or pleiotropy for human disease genes on multiple anatomical systems. This phenotypic effect across systems might arise from the complex clinical symptoms of rare diseases that manifests in multiple organs, tissues, or systems, which could indicate the levels of pleiotropy [38–40]. Hence, the comprehensive and deep phenotyping offered by HPO delivers a more systematic perspective on the functional roles of human disease genes, compared to the commonly used functional inferences based on human gene expression profile or *in vitro* screening. Interestingly, we discovered a significant negative correlation between the median Ka/Ks ratios and the number of affected anatomical systems in disease genes (the Pearson correlation coefficient ρ = −0.83, *p* = 0.0053). This implies that disease genes exhibiting higher pleiotropy, impacting multiple anatomical systems, are subject to more stringent evolutionary constraints compared to genes with low pleiotropy (Fig. 1e).

### Disease gene emergence rate per million years is similar across macroevolutionary epochs.

**Figure 2.**
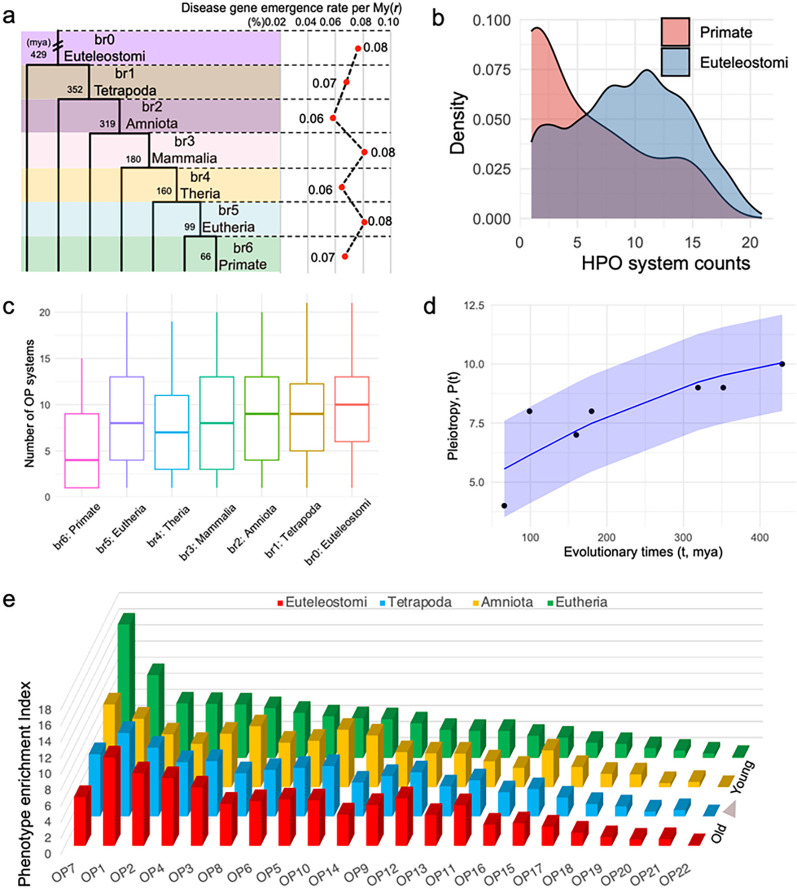
Disease gene emergence rates, phenotypic system coverage, and disease phenotype enrichment index (PEI) along evolutionary age groups **(a)** The disease-gene emergence rate per million years (*r*) across evolutionary epochs **(b)** Density distnbutions showcase the numbers of affected organ phenotypic systems (OPs) for genes originated at primate and Euteleostomi stage (c) Boxplot distributions showcase the numbers of affected organ phenotypic systems (OPs) for genes grouped by their evolutionary age (median values are 4. 8, 7, 8, 9. 9. 10, from left to right) (**d**) The nonlinear least squares (NLS) regression between pleiotropy score (P) and evolutionary times *t* with the logistic growth function ($P(t)=\frac{P_{\_}max}{1+\frac{P\_max=P_{\_}0}{P_{-}0}e^{-k(t)}}$, *K* = 1–66, *p* = 0 000787, 95% confidence interval is shown shade *P_max* and *P_0* are empirical medians 10 and 4, respectively) (**e**) The distribution of age and phenotype for the phenotype enrichment index (PEI) The bar plots colored differently, represent various age epochs, namely Euteleostomi, Tetrapoda. Amniota, and Euthena, in ascending order of age The organ phenotypes (OP) are displayed on the horizontal axis and defined in Figure 1c The standard deviations of PEI are 3 67 for Euthenan epochs and approximately 2 79 for older epochs.

To comprehend whether different evolutionary epochs have different emergence rate for disease genes, we assessed the disease gene emergence rate per million years across macroevolutionary stages from Euteleostomi to Primate (*μ_da_*). Considering the sampling space variations at different age group, we calculated *μ_da_* as the fraction of disease genes per million years at each stage (Fig. 2a). Although the proportions of disease genes were found to gradually increase from young to old age groups (Fig. 1a), the rate *μ_da_* is nearly constant ~ 0.07% per million years for different age groups (Fig. 2a). This constant disease gene emergence rate suggests a continuous and similar fraction of genes evolving to have significant impacts on health. Using the recently reported average human generation time of 26.9 years [41], the most updated number of coding genes (19,831 based on Ensembl v110), and assuming the simplified monogenic model [42], we estimated the number of casual genes for rare diseases per individual per generation (*μ_d_*) as 3.73 × 10^−4^ (= 19,831 × 26.9 × 0.07 × 10^−8^). Using this rate, we can derive the rare disease prevalence rate (*r_RD_* = 10,000 × *μ_d_*), which equates to approximately 4 in 10,000 individuals. This prevalence agrees remarkably well with the EU definition of rare disease rate prevalence of 5 in 10,000 people [43]. The constant parameter highlights the idea that young genes continually acquire functions vital for human health, which agrees with previous observations of young genes and their importance in contributing to phenotypic innovations [44–46].

### Young genes are highly enriched into phenotypes of the reproductive and nervous system.

Despite the nearly constant integration of young genes (Fig. 2a), it remains uncertain if gene age could influence disease phenotypic spectrums (or pleiotropy). The overall distribution of OP system counts for disease genes (Supplementary Fig. 2) is similar with the distribution of gene expression breath across tissues (Supplementary Fig. 3a-3c). The distribution for the numbers of OP systems showed that young genes have lower peak and median values than older genes (Fig. 2b–2c). This pattern was consistent with the results that younger genes tend to express in a limited range of tissues, while older genes exhibit a broader expression profile (Supplementary Fig. 3d), which also aligns with previously reported expression profiles [11, 47–49]. We found an increasing trend for the median numbers of OP systems from young to old evolutionary epochs (Fig. 2c). Interestingly, the increase rates $\left(\frac{\Delta OP\_median}{\Delta t}\right)$ are higher at the younger epochs than other older ones (0.12/mya at Eutherian stage vs. 0.05/mya at older stages on average, Supplementary table 4a), suggesting a non-linear and restricted growth model for the level of pleiotropy. We applied a logistic growth function and observed a significant pattern: as evolutionary time increases, the level of pleiotropy rises (Fig. 2d). Moreover, the model demonstrates a diminishing marginal effect, indicating that the rate of increase in pleiotropy slows down as evolutionary time continues to grow. This pattern suggests that pleiotropy is initially lower in new genes but increases at a faster rate compared to older genes. In addition, the higher pleiotropy in older genes is attributed to the cumulative effects over evolutionary history, rather than being inherently high from the outset.

To understand a finer-scale pattern of disease phenotypes for young and old genes, we introduced a metric of the disease phenotype enrichment index (PEI), which accounts for the range of phenotypes on multiple systems (see method for details). Our findings revealed that the most ancient genes, specifically from the Euteleostomi and Tetrapoda periods, had the strongest PEI association with the nervous system (OP1). Conversely, young genes from Amniota and Eutheria epochs tend to display the highest PEI for disease phenotypes of the genitourinary system (OP7) and the nervous system (OP1), with the former showing a 38.65% higher PEI than the latter (Fig. 2e, Supplementary table 4). Among the 22 disease phenotype systems, only the reproductive system (OP7) was unique in showing a steady rise in PEI from older epochs to younger ones (Fig. 2e). There were smaller variations in PEI for the older epochs when compared to the more recent Eutheria epoch (~ 2.79 vs. 3.67), hinting that older disease genes impact a greater number of organ systems, as also shown in Fig. 2c. This finding is consistent with the “out-of-testis” hypothesis [45], which was built on many observations where the expression patterns of young genes are limited to the testes and can have vital roles in male reproduction. As genes evolve over time, their expression tends to broaden potentially leading to increased phenotypic effects that impact multiple organ systems.

Apart from the reproductive system (OP7), we found that the nervous system (OP1) showed the second highest PEI for Eutherian young disease genes (Fig. 2e). Moreover, 42% of the 19 Primate-specific disease genes with diseases affecting the nervous system (OP1) correlate with phenotypes involving brain size or intellectual development (*CFC1, DDX11, H4C5, NOTCH2NLC, NOTCH2NLA, NPAP1, RRP7A,* and *SMPD4*. Supplementary table 2 and Discussion), consistent with the expectation of previous studies based on gene expression [29]. Furthermore, young genes emerging during the primate stage are connected to disease phenotypic enrichment in other adaptive systems, particularly in the HPO systems of the head, neck, eyes, and musculoskeletal structure (Fig. 2e). Overall, the Primate-specific disease genes could impact phenotypes from both reproductive and non-reproductive systems, particularly the genitourinary, nervous, and musculoskeletal systems (Supplementary table 2), supporting their roles in both sexual and adaptive evolution.

### Sex chromosomes are enriched for disease-associated genes.

**Figure 3:**
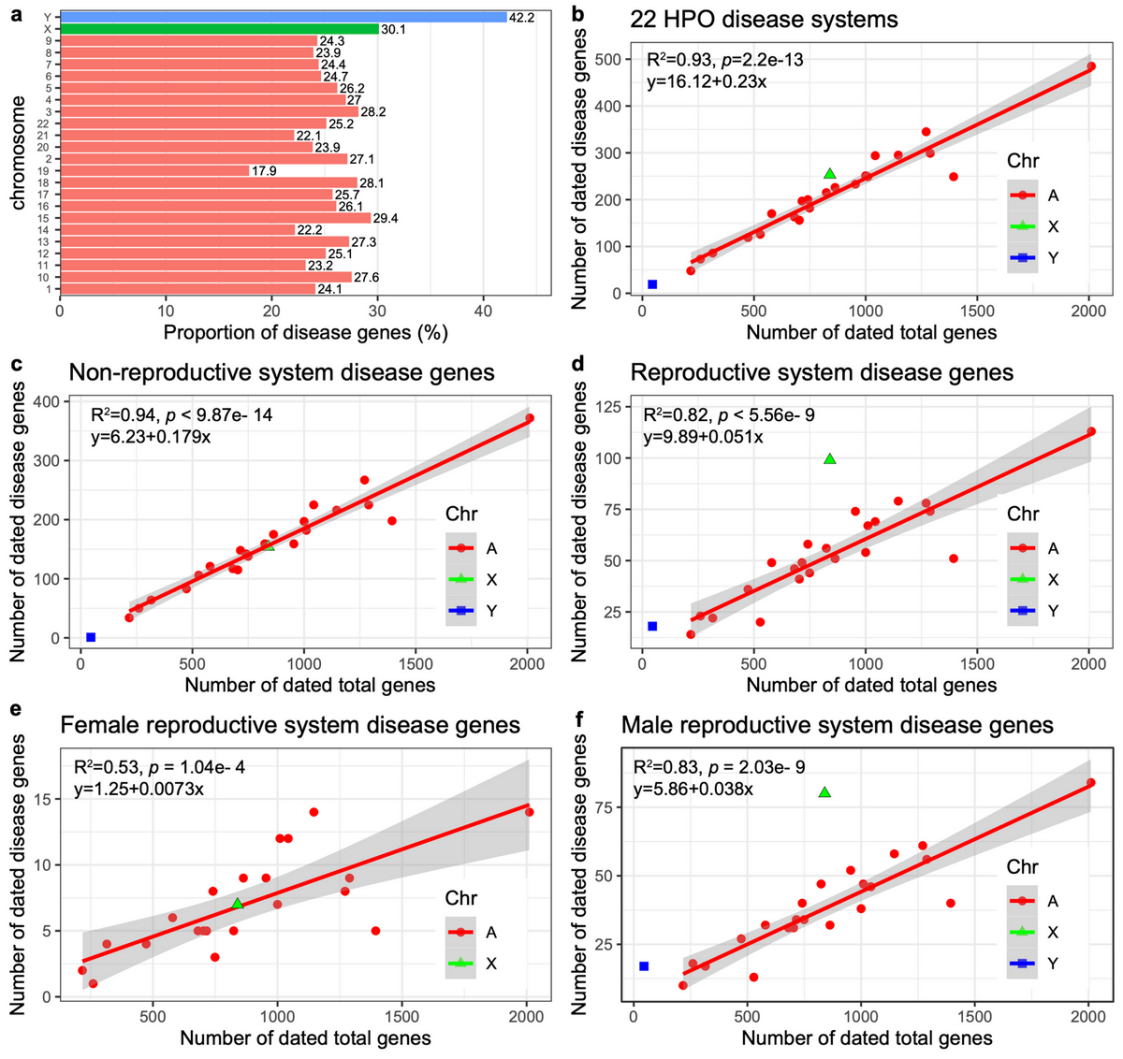
(**a**) The proportions of disease genes across chromosomes. The pink bars are the autosomes, while green and blue indicate the sex chromosome of X and Y respectively. The proportions (%) for different chromosomes are shown above bars. (**b**) The linear regression plotting of disease gene counts against the numbers of total genes with age information on chromosomes. (**c**) The number of genes related to the abnormality of non-genitourinary system (non-reproductive system) are plotted against all protein-coding genes on chromosomes with gene age information. (**d**) The number of genes related to the abnormality of genitourinary system (the reproductive system) are plotted against all protein-coding genes on chromosomes with gene age information. (**e**) Linear regression of dated disease gene counts against the total numbers of dated gene on chromosomes for female-specific reproductive disease genes. (**f**) Linear regression of dated disease gene counts against the total numbers of dated gene on chromosomes for male-specific reproductive disease genes. The autosomal linear models are shown on the top left corner. Note: All linear regression formulas and statistics pertain only to autosomes. “A”, “X”, and “Y” indicate autosomes, X and Y chromosomes, respectively.

Young gene duplicates with a bias toward male expression show chromosomal shifts between sex chromosomes and autosomes [50]. This movement might be an adaptation to address sexual conflicts in gamete formation or to avoid meiotic sex chromosome inactivation (MSCI) in spermatogensis [50–54]. Considering the rapid concentration of the youngest disease genes in the reproductive system (Fig. 2e, OP7), we hypothesized that disease genes related to various organs or tissues could have skewed chromosomal distributions. First, we examined the distribution of all disease genes and found a distinct, uneven spread across chromosomes (Fig. 3a and Supplementary table 5). The X and Y chromosomes have more disease genes than autosomal ones. While autosomes have a linear slope of 0.23 (Fig. 3b, *R*^2^ = 0.93; *p* = 2.2 × 10^–13^), the Y chromosome’s disease gene proportion is 82.61% higher at 0.42. Meanwhile, the X chromosome’s proportion is 30.43% more than autosomes, sitting at 0.301.

To understand if the differences between sex chromosomes and autosomes relate to reproductive functions, we divided disease genes into reproductive (1285 genes) and non-reproductive (3661 genes) categories (See Supplementary tables 6 and 7). By fitting the number of disease genes against all dated genes on chromosomes, we observed that the X chromosome exhibited a bias towards reproductive functions. Specifically, the X chromosome had slightly fewer disease genes affecting non-reproductive systems compared to autosomes (excess rate − 1.65%, observed number 154, expected number 156.59). In contrast, the X chromosome displayed a significant surplus of reproductive-related disease genes (observed number 99, expected number 52.73, excess rate 87.75%, *p* < 5.56e-9) (Fig. 3d). This result highlights the prominent difference in functional distribution between the X chromosome and autosomes, which might be attributed to the X chromosome’s unique role in reproductive functions. Given the sex-imbalanced mode of inheritance for the X chromosome, theoretical models have predicted that purifying selection would remove both dominant female-detrimental mutations and recessive male-detrimental mutations [55, 56]. We determined that the ratio of male to female reproductive disease genes (Mdisease/Fdisease or *α_d_*) is considerably higher for the X chromosome (80/9 = 8.89) than for autosomes on average (38/21 = 1.81, odds ratio = 16.08, 95% CI: 6.73–38.44, *p* < 0.0001). This suggests a disproportionate contribution of disease genes from the male hemizygous X chromosome compared to the female homozygous X. Thus, our analysis indicates that the abundance of disease genes on the X chromosome compared to autosomes might largely stem from male-specific functional effects. These data also hint that the overrepresentation of disease genes on the X chromosome is driven primarily by the recessive X-linked inheritance affecting male phenotypes rather than the dominant X-linked effect that impacts both genders.

### Sexual selection drives the uneven chromosomal distribution of reproductive disease genes.

To determine which gender might influence the biased distribution of reproductive-related genes on different chromosomes, we focused on genes specific to male and female reproductive disease. Based on the HPO terms of abnormalities in reproductive organs and gene age dating, we retrieved 154 female-specific and 945 male-specific disease genes related to the reproductive system with age dating data (Supplementary table 5 and 6). Through linear regression analysis, we assessed the number of gender-specific reproductive disease genes against the total counted genes for each chromosome. We observed strikingly different patterns that are dependent on gender and chromosomes.

For female reproductive disease genes, the X chromosome did not differ from autosomes, adhering to a linear autosomal pattern (*R*^2^ = 0.53, *p* = 1.04e-4, Fig. 3e). However, when examining male reproductive disease genes, the X and Y chromosomes starkly stood out compared to autosomes, which followed a linear pattern (*R*^2^ = 0.82, *p* = 5.56e-9, Fig. 3f). The X chromosome held an 111.75% more male reproductive genes than expected. Moreover, compared to autosomes (averaging 38/853), the sex chromosomes, Y (17/45) and X (80/840), demonstrated significantly higher ratios of male reproductive disease genes, with odds ratios of 8.48 (95% CI: 4.45–16.17, *p* < 0.0001) and 2.14 (95% CI: 1.44 to 3.18, *p* = 0.0002), respectively. On the X chromosome, the fraction of male reproductive genes was 10.43 times greater than that of female reproductive genes (80/840 vs. 7/840). This observation is consistent with the “faster-X hypothesis”, where purifying selection is more effective in eliminating recessive deleterious mutations on the X chromosome due to the male hemizygosity of the X chromosome [55, 56]. Interestingly, we also observed a male-bias in reproductive disease gene density on autosomes, where the slope of the autosomal linear model for males was approximately 4.21 times steeper than for female (0.038 vs. 0.0073) (Fig. 3e and 3f). Thus, our observed excess of male reproductive disease genes is not caused solely by the “faster-X” effect. It might also be influenced by the “faster-male” effect, postulating that the male reproductive system evolves rapidly due to heightened sexual selection pressures on males [57].

### Excess of young genes with male reproductive disease phenotypes

**Figure 4:**
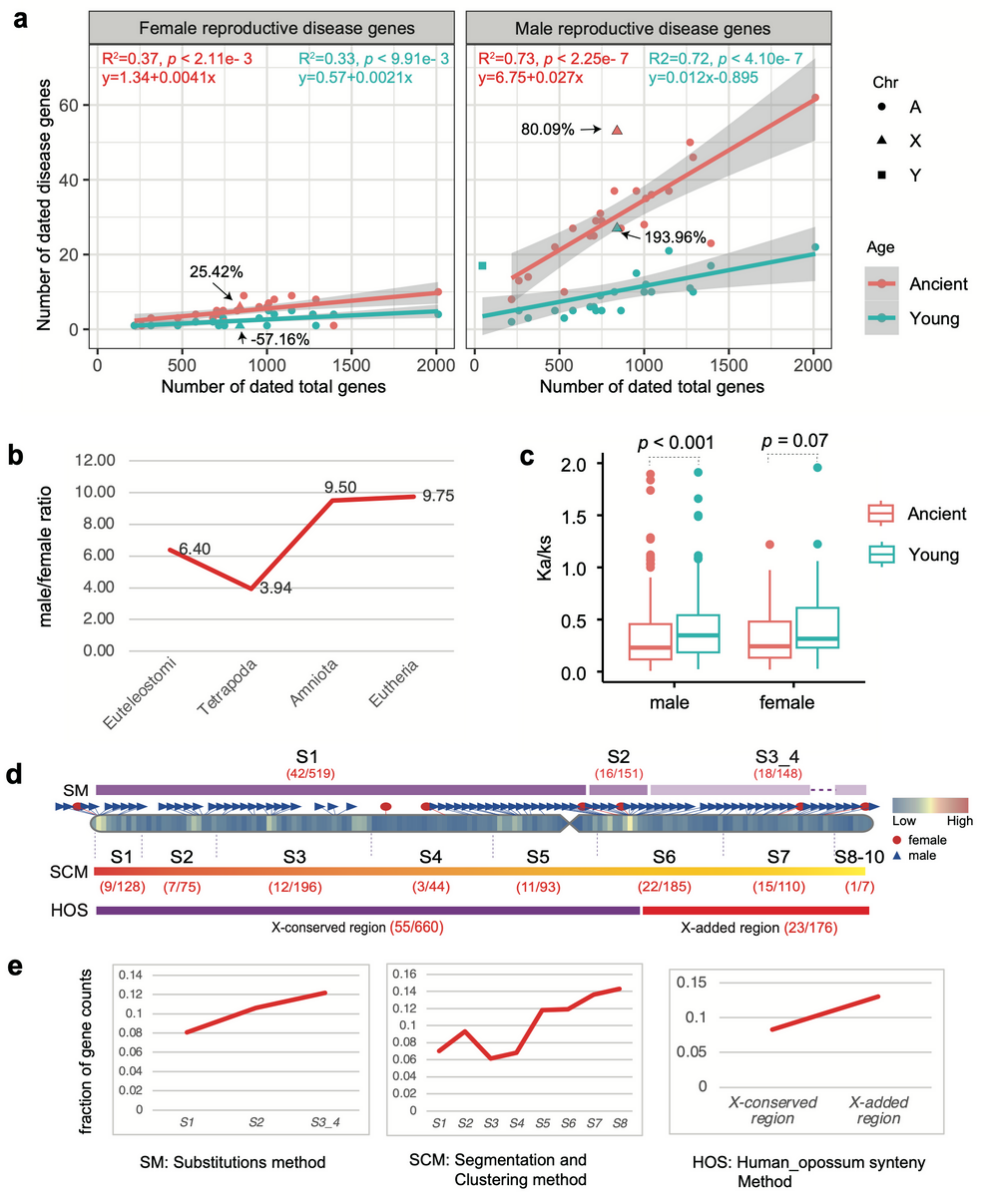
(**a**) The numbers of female-specific (left) and male-specific reproductive disease genes (right) are plotted against all protein-coding genes with gene ages on chromosomes. The linear formulas fitted for autosomal genes at ancient (Euteleostomi) and younger (post-Euteleostomi) stages are shown in red and blue, respectively. (**b**) The ratios of male to female reproductive disease gene numbers () across four evolutionary epochs. (**c**) The comparison of selection pressure (human-chimpanzee pairwise Ka/Ks) for sex-specific reproductive disease genes between the ancient (stage Euteleostomi) and younger (post-Euteleostomi) epochs. Only the autosomal comparison is shown, with p value from the Wilcoxon test. (**d**) The numbers of male-specific reproductive disease genes (*m*) and the background genes (*b*) within the subregions from old to young on the X chromosome are provided, with the numbers displayed within round brackets for each subregion (*m/b*). SM, SCM, and HOS denote three classification methods for X chromosome structure: the substitutions method [60, 62], the segmentation and clustering method [59], and the synteny method (orthologous gene order conservation between human and opossum). (**e**) The fraction of disease genes with male-specific reproductive disease phenotypes within each stratum or subregion, as illustrated in (**d**), is presented. The gene coordinates have been updated based on the hg38 reference with liftover tool. “A”, “X”, and “Y” indicate autosomes, X and Y chromosomes, respectively.

While we observed a male-bias in reproductive disease genes, the influence of gene ages as a factor on this excess remains unclear. We compared gene distribution patterns between older (or ancient, stage Euteleostomi) and younger (post-Euteleostomi) stages. For female-specific reproductive disease genes, the X chromosome has an excess of ancient genes but a deficiency of young genes (25.42% vs. −57.16%, Fig. 4a). Conversely, for male-specific reproductive disease genes, younger genes exhibited a higher excess rate than ancient genes (193.96% vs. 80.09%) (Fig. 4a). These patterns suggest an age-dependent functional divergence of genes on the X chromosome, which is consistent with gene expression data. The X chromosome is “masculinized” with young, male-biased genes and old X chromosomal genes tend to be “feminized,” maintaining expression in females but losing it in males [52]. On autosomes, the linear regression slope values were higher for male reproductive disease genes than for female ones, both for ancient (0.027 vs. 0.0041) and young genes (0.012 vs. 0.0021) (Fig. 4a). The ratio of male to female reproductive disease gene counts (*αd*) showed a predominantly male-biased trend across epochs, with a higher value in the most recent epoch of Eutheria (9.75) compared to the ancient epochs Euteleostomi and Tetrapoda (6.40 and 3.94, Fig. 4b). Selection pressure comparison between young and ancient genes revealed no significant difference for female-specific reproductive disease genes, but significant difference for male-specific ones (Fig. 4c, the Wilcoxon rank-sum test, *p* < 0.0001), indicating that young genes under sexual selection have less evolutionary constraints than older ones (median Ka/Ks ratio 0.35 vs. 0.23).

Structurally, the eutherian hemizygous X chromosome comprises an ancestral X-conserved region and a relatively new X-added region [58]. The ancestral X-conserved region is shared with the marsupial X chromosome, whereas the X-added region originates from autosomes (Fig. 4d). To understand which human X chromosome regions might contribute differentially to human genetic disease phenotypes, we compared genes within the X-conserved and X-added regions, based on previous evolutionary strata and X chromosome studies [59–61]. After excluding genes on X-PAR (pseudoautosomal regions) regions (Ensembl v110), we found that the proportion of male-specific reproductive disease genes in X-added region (13.07%, 23/176) exceeds that in the X-conserved region (8.33%, 55/660) (Fig. 4d and 4e, Supplementary table 7). Moreover, analyses of the evolutionary strata, which relies on substitutions method (Lahn and Page 1999; McLysaght 2008) and the segmentation and clustering method (Pandey et al. 2013), consistently showed higher fractions of male-specific reproductive disease genes in younger evolutionary strata than in older ones (Fig. 4e). These observations indicate that, on the X chromosome, young genes could be more susceptible to the forces of sexual selection than old genes, despite their nearly identical hemizygous environment.

## Discussion

### The underestimated roles of young genes in human biomedically important phenotypes and innovations.

After the discovery of the first disease gene in 1983, which was based on linkage mapping for a Huntington’s disease with pedigree [62], there has been a rapid advancement in medical genetics research. As of now, this field has identified approximately 20% of human genes (~ 4000–5000 genes) with phenotypes of the rare or “orphan” diseases [7, 63–74]. In our study, we utilized the latest disease gene and clinical phenotype data from HPO annotations [33] and incorporated synteny-based gene age dating to account for new gene duplication events [32]. Contradicting the prior belief that only a tiny fraction of Eutherian young genes are related to diseases [16], our synteny-based gene age dating reveals almost a tenfold increase, suggesting the substantial role of young genes in human biomedical phenotypes. Despite previous debates on the selective pressure of disease genes [16, 75–77], our comparative analyses of Ka/Ks ratios between humans and primates consistently show stronger purifying selection on disease genes than non-disease genes, indicating evolutionary constraints to remove harmful mutations. The epoch-wise estimates of the emergence rate of disease genes per million years reveal a steady integration of genes into disease phenotypes, which supports Haldane’s seminal 1937 finding that new deleterious mutations are eliminated at the same rate they occur [78, 79].

### Young genes rapidly acquire phenotypes under both sexual and natural selection.

The chromosomal distribution of all disease genes shows the excess of disease genes in X chromosome (Fig. 3), which supports the “faster-X effect” [55, 56], that male X-hemizygosity could immediately expose the deleterious X chromosome mutations to purifying selection. Conversely, the X-chromosome inactivation (XCI) in female cells could lessen the deleterious phenotypes of disease variants on the X chromosome [80]. The X chromosome excess of disease genes is attributed predominantly to that of the male reproductive disease genes (Fig. 3). This male-specific bias was not limited to the sex chromosome but also detectable in autosomes (Fig. 3). These findings align with the “faster-male” effect, where the reproductive system evolves more rapidly in males than in females due to heightened male-specific sexual selection [57]. Intriguingly, of the 22 HPO systems, young genes are enriched in disease phenotypes affecting the reproductive-related system. As genes age, there’s a marked decline in both PEI (phenotype enrichment index) and (the male-to-female ratio of reproductive disease gene numbers). These patterns are consistent with the “out of testis” hypothesis [45], which describes the male germline as a birthplace of new genes due to factors including the permissive chromatin state and the immune environment in testis [81, 82]. The “out of testis” hypothesis predicts that genes could gain broader expression patterns and higher phenotypic complexity over evolutionary time [82]. Consistently, we observed a pattern where older sets of disease genes have phenotypes over a much broader anatomical systems compared to younger genes which tend to impact limited systems. The strong enrichment of male reproductive phenotypes for young genes is also consistent with findings from model species that new genes often exhibit male-reproductive functions [50, 83], in both *Drosophila* [53, 83, 84] and mammals [51, 85]. Some new gene duplicates on autosomes are indispensable during male spermatogenesis, to preserve male-specific functions that would otherwise be silenced on the X chromosome due to the meiotic sex chromosome inactivation (MSCI) [51, 52, 85].

Apart from the reproductive functions, new genes are also enriched for adaptive phenotypes. Previous transcriptomic studies indicate that new genes have excessive upregulation in the human neocortex and under positive selection [29]. The brain size enlargement, especially the neocortex expansion over ~ 50% the volume of the human brain, ranks among the most extraordinary human phenotypic innovations [29, 86]. Here, we found that at least 42% of primate-specific disease genes affecting the nervous systems could impact phenotypes related to brain size and intellectual development. For example, *DDX11* is critical in pathology of microcephaly [87–90]. The *NOTCH2NLA, NOTCH2NLB*, and *NOTCH2NLC* may promote human brain size enlargement, due to their functions in neuronal intranuclear inclusion disease (NIID), microcephaly, and macrocephaly [91–93]. The *RRP7A* is also a microcephaly disease gene evidenced from patient-derived cells with defects in cell cycle progression and primary cilia resorption [94]. The defects of *SMPD4* can lead to a neurodevelopmental disorder characterized by microcephaly and structural brain anomalies [95]. The *SRGAP2C* accounts for human-specific feature of neoteny and can promote motor and execution skills in mouse and monkey model [96–98]. The de novo gene *SMIM45* [99] associates with cortical expansion based on extensive models [31].

New genes were also found with enrichment in other adaptive phenotypes, particularly involving the head and neck, eye, and musculoskeletal system. Some examples of these primate-specific disease genes encompass *CFHR3* associated with macular degeneration [100], *SMPD4* with the retinopathy [101], *TUBA3D* with the keratoconus [102], *OPN1MW* with loss of color vision [103, 104], *YY1AP1* with Fibromuscular dysplasia [105], *SMN2* with the Spinal Muscular Atrophy [106], *GH1* with defects in adult bone mass and bone loss [107], *KCNJ18* with thyrotoxicosis complicated by paraplegia and hyporeflexia [108], *TBX5* with the cardiac and limb defects of Holt-Oram syndrome [109, 110], and *DUX4* with muscular dystrophy [111]. Additionally, some other specific functions have also been reported for these young genes. For example, the Y chromosome gene *TBL1Y* could lead to male-specific hearing loss [112]. The *TUBB8* defects could lead to complete cleavage failure in fertilized eggs and oocyte maturation arrest [113–115]. Interestingly, a previous case study on mice also shows the role of *de novo* genes on female-specific reproductive functions [116]. These emerging studies independently support the importance of new genes in phenotypic innovation and sexual selection, refuting previous assumptions that new genes contribute little to phenotypic innovation [117].

### New genes underlying rapid phenotypic innovations: low pleiotropy as a selective advantage.

**Figure 5.**
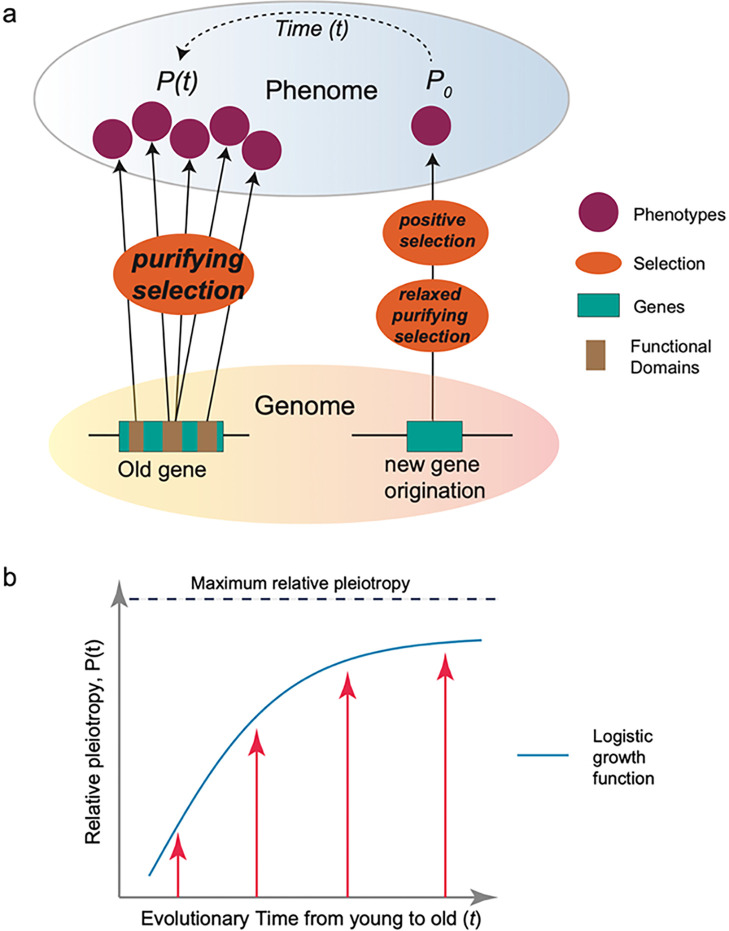
**(a)** The ‘pleiotropy-barrier model’ posits that new genes evolve adaptively more quickly It suggests that older genes undergo stronger punfying selection because their multiple functions (usually adverse pleiotropy) act as a barrier to the uptake of mutations that might otherwise be beneficial for novel phenotypes **(b)** The logistic function between relative pleiotropy P(*t*) and evolutionary time *t. $P(t)=\frac{P\_\;\text{max}}{1+e^{-kt}}$* where *P_max* represents the maximum relative pleiotropy The *k* is the growth rate parameter, which controls how quickly the phenomenon approaches the maximum value A higher k value means faster growth initially.

Our findings raise the question of why new genes can quickly enrich into phenotypic traits that are crucial for both sexual evolution and adaptive innovation. This question could not be fully addressed by previous hypotheses. The “out of testis” theory, as well as the “male-driven,” “faster-X,” and “faster-male” theories, do not offer specific predictions regarding the propensity of new or young genes to be involved in adaptive traits. Here, we proposed a “pleiotropy-barrier” model to explain the relationship between innovation potential and gene ages (Fig. 5a). The evidence of extensive pleiotropy was found early in the history of genetics [118–120]. It is established that young genes exhibit higher specificity and narrower expression breadth across tissues [48]. In this study, we used a broader definition of pleiotropy to understand phenotype evolution [38, 121–123]. We reveal a pattern that older genes tend to impact more organs/systems, while young genes display phenotype enrichment in specific organs (Fig. 2c). Therefore, both phenotype pattern and expression trend across evolutionary epochs suggest lower pleiotropy for young genes, compared to the progressively higher pleiotropy observed in older genes.

Numerous theoretical and genomic studies have revealed that pleiotropy impedes evolutionary adaptation (a so-called ‘cost of complexity’) [118, 124–127], while low pleiotropy could foster more morphological evolutions [128, 129]. The inhibitory effect of pleiotropy on novel adaptation aligns with our observations of the strong purifying selection on both high extent of pleiotropy [124, 125] and expression breadth [130]. As expected, we observed that multi-system genes and older genes, which exhibit higher pleiotropy, undergo stronger purifying selection (Fig. 1b–1e). This evolutionary constraint suggests a restricted mutation space to introduce novel traits for old genes due to the “competing interests” of multifunctionality (Fig. 5). The inhibitory pressure could also reduce genetic diversity due to background selection [131]. The evolution of new genes, especially gene duplicates, serves as a primary mechanism to mitigate pleiotropic effects through subfunctionalization and neofunctionalization [132, 133] and avoid adverse pleiotropy in ancestral copies [134]. The tissue-specific functions of new genes, as a general pattern in numerous organisms, could circumvent the adaptive conflicts caused by the multifunctionality of parental genes [135]. The reduced pleiotropy in young genes could thereby allow for a more diverse mutational space for functional innovation without triggering unintended pleiotropic trade-offs [136].

The “pleiotropy-barrier” model predicts that the capacity for phenotypic innovation is limited by genetic pleiotropy under nature selection (Fig. 5a). Over evolutionary time, the pleiotropy increase follows a logistic growth pattern, where the speed of growth could be higher for younger genes but lower for older genes (Fig. 5b). The multifunctional genes could encounter an escalating “barrier” toward the pleiotropy maximum. This barrier arises because more functions necessitate stronger selective constraints, which could in turn reduce mutational space of beneficial mutations for novel phenotypes. In contrast, low or absent pleiotropy in new genes allows for a higher and tunable mutation space under the relaxed purifying selection. The permissive environment provides a fertile ground for beneficial mutations to appear with novel functions. Such innovations, initially as polymorphisms within a population, can become advantageous phenotypes and ready responder in certain environment under positive selection. Therefore, young genes, with lower pleiotropic effect as a selective advantage, not only spurs molecular evolution under sexual and natural selection but, from a medical standpoint, also are promising targets for precise medicine, warranting deeper investigation.

## Conclusion

In this study, we unveil a remarkable pattern of new gene evolution with vital pathogenic functions shaped by the non-neutral selection. Although the ratio of genes associated with health-related functions per million years remains relatively consistent across macroevolutionary epochs, we note an enrichment pattern of disease systems for young genes. Importantly, young genes are preferentially linked to disease phenotypes of the male reproductive system, as well as systems that undergone significant phenotypic innovations in primate or human evolution, including the nervous system, head and neck, eyes, and the musculoskeletal system. The enrichment of these disease systems points to the driving forces of both sexual selection and adaptive evolution for young genes. As evolutionary time progresses, older genes display fewer specialized functions compared to their young counterparts. Our findings highlight that young genes are likely the frontrunners of molecular evolution, being actively selected for functional roles by both adaptive innovation and sexual selection, a process aided by their lower pleiotropy. Therefore, young genes play a pivotal role in addressing a multitude of questions related to the fundamental biology of humans.

## Materials and Methods

### Gene age dating and disease phenotypes

The gene age dating was conducted using an inclusive approach. For autosomal and X chromosomal genes, we primarily obtained gene ages (or branches, origination stages) from the GenTree database [32, 52] that is based on Ensembl v95 of human reference genome version hg38 [137]. We then trans-mapped the v95 gene list of GenTree into the current release of Ensembl gene annotation (v110). The gene age inference in the GenTree database relied on genome-wide synteny and was based on the presence of syntenic blocks obtained from whole-genome alignments between human and outgroup genomes [11, 32, 52]. The most phylogenetically distant branch at which the shared syntenic block was detected marks the time when a human gene originated. In comparison to the method based on the similarity of protein families, namely the phylostratigraphic dating [138], this method employed in GenTree is robust to recent gene duplications [32], despite its under-estimation of the number of young genes [87]. We obtained gene age for human Y genes through the analysis of 15 representative mammals [139]. Notably, Y gene ages are defined as the time when these genes began to evolve independently of their X counterpart or when they translocated from other chromosomes to the Y chromosome due to gene traffic (transposition/translocation) [139]. For the remaining Ensembl v110 genes lacking age information, we dated them using the synteny-based method with the gene order information from ENSEMBL database (v110), following the inference framework of GenTree [32]. These comprehensive methods resulted in the categorization of 19,665 protein-coding genes into distinct gene age groups, encompassing evolutionary stages from Euteleostomi to the human lineage, following the phylogenetic framework of the GenTree database. The HPO annotation used in this study for phenotypic abnormalities contains disease genes corresponding to 23 major organ/tissue systems (09/19/2023, https://hpo.jax.org/app/data/annotations). After filtering out mitochondrial genes, unplaced genes, RNA genes, and genes related to neoplasm ontology, we obtained with gene ages and phenotypic abnormalities (across 22 categories) for 4946 protein-coding genes. The reproductive system disease genes were retrieved from the “phenotype to genes.txt” file based on “reproduct”, “male”, “female” keywords (neoplasm-related items were removed).

### Ka/Ks ratio

Ka/Ks is widely used in evolutionary genetics to estimate the relative strength of purifying selection (Ka/Ks < 1), neutral mutations (Ka/Ks = 1), and beneficial mutations (Ka/Ks > 1) on homologous protein-coding genes. Ka is the number of nonsynonymous substitutions per non-synonymous site, while Ks is the number of synonymous substitutions per synonymous site that is assumed to be neutral. The pairwise Ka/Ks ratios (human-chimpanzee, human-bonobo, and human-macaque) were retrieved from the Ensembl database (v99) [137], as estimated with the Maximum Likelihood algorithm [140].

### Disease gene emergence rate per million years (r)

To understand the origination tempo of disease genes within different evolutionary epochs, we estimated the disease gene emergence rate per million years r for disease genes, which is the fractions of disease genes per million years for each evolutionary branch. The calculating is based on the following formula:

$$r_{i}=\frac{O_{i}}{A_{i}T_{i}}$$

where $r_{i}$ represents the phenotype integration index for ancestral branch i. The $O_{i}$ indicates the number of disease genes with organ phenotypes in ancestral branch i. The denominator $A_{i}$ is the number of genes with gene age information in branch i. The $T_{i}$ represents the time obtained from the Timetree database (http://www.timetree.org/) [141].

### Pleiotropic modeling with logistic growth function

For each evolutionary epoch (t), we estimated the median numbers of OP systems that genic defects could affect, which serve as the proxy of pleiotropy over evolutionary time (P(t)) for regression analysis. The logistic growth function was used to fit the correlation with the Nonlinear Least Squares in R.

### Phenotype enrichment along evolutionary stages

The phenotype enrichment along evolutionary epochs was evaluated based on a phenotype enrichment index (PEI). Specifically, within “gene-phenotype” links, there are two types of contributions for a phenotype, which are “one gene, many phenotypes” due to potential pleiotropism as well as “one gene, one phenotype”. Considering the weighting differences between these two categories, we estimated the $PEI_{\left(i,j\right)}$ or a given phenotype $\left(p_{i}\right)$ within an evolutionary stage $\left(br_{j}\right)$ with the following formula.

$${PEI}_{\left(i,j\right)}=\frac{\sum_{i=1}^{n}\;\;\frac{1}{m}}{\sum_{J=1}^{l}\;\;\sum_{i=1}^{n}\;\;\frac{1}{m}}$$

The m indicates the number of phenotype(s) one gene can affect, n represents the number of genes identified for a given phenotypes, and l is number of phenotypes within a given evolutionary stage. Considering the genetic complexity of phenotypes, the enrichment index (*PEI*) firstly adjusted the weights of genes related to a phenotype with the reciprocal value of m, *i.e*., $\frac{1}{m}$. Thus, the more phenotypes a gene affects, the less contributing weight this gene has. Then, we can obtain the accumulative value (*p*) of the adjusted weights of all genes for a specific phenotype within an evolutionary stage. Because of the involvement of multiple phenotypes within an evolutionary stage, we summed weight values for all phenotypes $\left(\sum_{J=1}^{l}\;p\right)$ and finally obtained the percentage of each phenotype within each stage $\left(\frac{p}{\sum_{J=1}^{l}\;p}\right)$ as the enrichment index.

### The linear regression and excessive rate

The linear regression for disease genes and total genes on chromosomes was based on the simple hypothesis that the number of disease genes would be dependent on the number of total genes on chromosomes. The linear regression and statistics were analyzed with R platform. The excessive rate was calculated as the percentages (%) of the vertical difference between a specific data point, which is the number of gene within a chromosome (n), and the expected value based on linear model (n-e) out of the expected value $\left(\frac{n-e}{e}\right)$.

### The X-conserved and X-added regions

The Eutherian X chromosome is comprised of the pseudoautosomal regions (PAR), X-conserved region, and X-added region. The regions of two PAR were determined based on NCBI assembly annotation of GRCh38.p13 (X:155701383–156030895 and X:10001–2781479). The X-boundary between X-conserved and X-added regions was determined with Ensembl biomart tool. The “one to one” orthologous genes between human and opossum were used for gene synteny identification. The X-conserved region is shared between human and opossum, while X-added region in human has synteny with the autosomal genes of opossum [61]. The “evolutionary strata” on X were based on previous reports of two methods: substitutions method and the Segmentation and Clustering method [59, 60, 142]. The coordinates of strata boundaries were up-lifted into hg38 genome with liftover tool (https://genome.ucsc.edu/cgi-bin/hgLiftOver).
